# Post-natal care: a vital chance to save mothers and infants! Exploring barriers and factors associated with it: a mixed study

**DOI:** 10.3389/fgwh.2023.1272943

**Published:** 2023-10-25

**Authors:** Bekem Dibaba Degefa, Gizu Tola Feyisa, Dagne Deresa Dinagde, Gemeda Wakgari Kitil, Agmasie Damtew Walle

**Affiliations:** ^1^ Department of Midwifery, College of Health Science, Mattu University, Mattu, Ethiopia; ^2^ Department of Health Informatics, College of Health Science, Mattu University, Mattu, Ethiopia

**Keywords:** postnatal care, factors, utilization, services, barriers

## Abstract

**Introduction:**

The most effective maternal health intervention for enhancing mother and baby survival is postnatal care, yet it is also the most neglected service in Ethiopia. Less is known about postnatal care despite earlier studies concentrating on pregnancy and delivery service utilization. Postnatal care is the subject of few national and local area studies. Therefore this research aims to evaluate postnatal care utilization and barriers and associated characteristics among women in Ilubabor Zone and Buno Bedele Zone.

**Methods:**

A mixed-methods study involving women who visited immunization clinics was conducted in Southwest Ethiopia. For the quantitative part, a cross-sectional survey was conducted between June 12 and July 12, 2022. The data collected through interviews was analyzed using SPSS version 26. An adjusted odds ratio (AOR) with a 95% confidence interval (CI) and *p*-value was constructed to evaluate the associations between postnatal care service utilization and explanatory variables. The usage of postnatal care services was determined to be significantly correlated with explanatory variables in multivariable logistic regression with a *p*-value less than 0.05. This qualitative study used two focused group discussions and two in-depth interviews to gather data from purposely selected mothers, and thematic analysis was used to analyze the data.

**Results and Discussion:**

A total of 422 participants with a 100% response rate were included in the analysis. 234 (55.5%) of these underwent postnatal checks. In the quantitative section, postnatal care counseling and appointment setting, counseling on danger signs, and prior postnatal care utilization all demonstrated a statistically significant association with the use of postnatal care services (AOR = 3.6, 95% CI (1.47–7.23)), [AOR = 2, 95% CI (1.05–3.64)], and [AOR = 3, 95% CI (1.36–58), respectively). At the qualitative level, it was determined that the themes of knowledge and access were obstacles to the use of postpartum care services. Generally this study revealed that the Ilubabor Zone and Buno Bedele Zone have a poor total PNC service utilization rate. Furthermore, ignorance, conventional wisdom, religious activity, distance from facilities, environmental exposure, and waiting time were identified as barriers to postnatal care service utilization. To optimize this service, all parties involved should address these factors.

## 1. Introduction

Globally, approximately 1.5 million mothers die after childbirth each year. In developing countries, 1 in 7 mothers die from pregnancy and childbirth particularly during the postnatal period. The global community has addressed women's health in the past few years. Reduced maternal mortality and universal access to reproductive health care were two objectives of the fifth Millennium Development Goal (MDG) for maternal health. However, it is believed that the MDG had a low success rate (1–4).

Ethiopia still has a high rate of maternal and newborn mortality, similar to other third-world nations. In Ethiopia, direct reasons such as hemorrhage, infection, obstructed labor, unsafe abortions, and high blood pressure, which typically happen during the postpartum period, account for over 80% of maternal mortality. In Ethiopia, severe obstetric hemorrhage is a leading cause of death. The fastest maternal killer is postpartum hemorrhage, which can kill even a healthy woman within 2 h if left untreated. To prevent this postpartum issue, the postnatal period, which is defined as the first six weeks following birth, is essential for the health and survival of both a woman and her infant. For monitoring delivery-related difficulties, the first two days following delivery are particularly important. Due to this, a postnatal visit is the ideal time to inform the mother of danger signs, and all women should have at least three to four postnatal visits, as advised by the WHO. If care is not given during this period, missed opportunities to promote healthy behaviors in mothers and newborns may end in death or impairment. The figures on postnatal service utilization are deficient for countries like Ethiopia, where 75% of women do not receive any postpartum care, followed by Bangladesh (73%), Nepal (72%), and Rwanda (71%). Other countries with sizable proportions of women who did not obtain any postpartum medical care include Burkina Faso (44%), Cambodia (46%), Haiti (55%), Kenya (46%), and Malawi (41%). Mali (49%), Nigeria (46.5%), Uganda (57%) and Zambia (41%) also showed comparable results. On average, nearly 40% of women did not receive a postpartum care checkup in the 30 countries studied (5–11).

Postnatal care obstacles and other factors have been found in prior work. Findings from studies conducted in Jabitena district, Amhara regional state, Northwest Ethiopia, Northern Ethiopia, and Wolkite, Ethiopia, as well as rural areas of Northern Ethiopia, Tigray, Ethiopia, Debre Tabor town, Northern Ethiopia, Hawassa Zuria, Northern Ethiopia at Adigirat town, Addis Ababa, in Sodo Zuria and Shebe Sombo Woreda, Jimma, revealed that the prevalence of postnatal service utilization ranges from 20.2% to 77.7% (12–30).

Some studies conducted in parts of Ethiopia assessed a number of barriers and factors associated with postnatal care service utilization. For instance, a community-based study in Northern Ethiopia found that women who delivered at health institutions were three times more likely to attend postnatal care services than those who delivered at home. Similarly, women who knew about the complications related to pregnancy/labor were more likely to use postnatal care services than those who did not. In addition, women were 4.6 times more likely to use postnatal care services if they were aware of them than if they were unaware. The number of children ever delivered, the mother's level of literacy, and radio listening frequencies were determined to be the drivers of PNC use in a study conducted in parts of Ethiopia. Other factors such as more than four pregnancies, wanted pregnancy, spontaneous vertex delivery, husband with secondary education, delivery with cesarean section, secondary education or above for the mother, a monthly household income of more than 1,500 ETB, planned and supported prior pregnancy, institutional delivery of last pregnancy, accessing health care, having a positive attitude towards the use of postnatal services, and having a living child born from the previous pregnancy show significant associations with postnatal service utilization (13, 14, 20–29).

The most effective maternal health intervention for enhancing mother and baby survival is postnatal care, yet it is also the most neglected service in Ethiopia. Less is known about postnatal care, despite earlier studies concentrating on pregnancy and delivery service consumption. Postnatal care is the subject of few national and local area studies. Therefore this study aims to evaluate postnatal care utilization, barriers and associated characteristics among women in Southwest Ethiopia who gave birth 10 weeks before the survey and who visited selected health centers for child immunizations up to 14 weeks following delivery in the of Ilubabor Zone and Buno Beadle Zone.

## 2. Methods and materials

### 2.1. Study area, design and period

A mixed methods study was carried out at health centers in Southwest Ethiopia, between June 12 and July 12, 2022. For the quantitative study cross-sectional study design was used whereas the qualitative study was carried out to triangulate the quantitative study. Ilubabor Zone has one referral hospital, one district hospital, and forty health centers that serve its residents whereas Buno Bedele has thirty two health facilities, 246 health posts, three operational hospitals, and one hospital that is currently being built.

### 2.2. Populations

For the quantitative part, the study population consisted of all women in the Ilubabor and Buno Bedele zones who gave birth 10 weeks before the survey and who visited health facilities for child immunizations up to 14 weeks following delivery and who met the inclusion criteria throughout the chosen time. For the qualitative part we selected mothers purposely from similar populations.

### 2.3. Inclusion and exclusion criteria

Women who visited the specified medical institutions between 10 and 14 weeks following delivery to have their child immunized and gave their informed consent were included.

### 2.4. Sample size determination, sampling technique, and procedures

With the following assumptions: *p* = 48%, coverage of postnatal care service utilization in urban areas (EDHS 2019), a 95% confidence level, a 5% precision, and a 10% non-response rate, the sample size was calculated using a single population proportion formula, and the final sample size was 422 for the quantitative part. Based on each health center's 6-month infant immunization rate, the total sample sizes were distributed among the health centers in a proportional manner. Throughout the study period, all eligible women in each health center were invited to participate in turn until the necessary sample size was reached. Ten women were chosen for the qualitative level using the purposive sampling method. From 72 health centers in the two zones 21 health centers were selected by systematic random sampling technique and the total sample size was distributed for each health center based on 6 months of client flow in the immunization clinic.

### 2.5. Data collection tool, procedure, and quality control

After reading the pertinent literature, the questionnaire for the quantitative portion was modified to address the study objectives. Then, 5% of the sample participants underwent pretesting outside the research location. Accordingly, the questionnaire was modified. Quantitative information was gathered via Open Data Kit (ODK) using standardized questionnaires that were pretested and administered by interviewers. During and after data collection, the lead investigators and supervisors evaluated and verified the questionnaire to ensure its accuracy and applicability. The quantitative level gathered information from new mothers who visited health clinics to have their children immunized and receive family planning. Data collectors received a half-day orientation from the supervisors overseeing the entire data collection process. The survey was written in English, translated into Afan Oromo, then back into English to ensure uniformity. Two weeks before the real data collection period, a pretest was conducted. Every day before entering the data, data collectors, supervisors, and the lead investigator verified all the information that had been obtained. In addition, non-respondents were counted for any incomplete surveys that missed >10% of the total response.

Data were gathered at the qualitative level using a semi-structured guide. To assure the quality of the data, the guide was first pretested on four women before the real data collection. The guide included major theme areas of knowledge about postnatal care service utilization, accessibility, financial constraints and traditional and religious barriers. Afan Oromo was used to translate the conceptual guide from English. The idea behind the guide was to move from general principles to precise specifics. In-depth interviews and focused group discussions were used for 48 and 50 min respectively to gather information from the participants. The modulator served as the principal investigator for the duration of the entire data collection process.

### 2.6. Study variables

#### 2.6.1. Dependent variable

Postnatal care service utilization.

#### 2.6.2. Independent variable

Socio-demographic characteristics: age, ethnicity, and religion, current state of marriage, monthly income, and occupation of the partner.

Obstetric and reproductive factors affecting mothers include gravidity, parity, abortion, number of living children, type of pregnancy (planned vs. unplanned, supported vs. unsupported), distance to health facility, mode of delivery, location of delivery, use of antenatal care, use of postpartum care in the past, and length of stay in the facility following delivery.

### 2.7. Operational definition

Utilization of postnatal care services: postnatal care (PNC) is the care given to the mother and their newborn baby immediately after the birth and for the first six weeks of life.

### 2.8. Data processing and analysis

After data collection, each questionnaire was examined for completeness and coding on a quantitative level. Epidata version 4.6 was used to enter the data, which was subsequently exported to SPSS (Statistical Package for Social Sciences, version 26) for analysis. Using variable logistic regression analysis, descriptive summaries, frequency, and percentages were employed to summarize the study variables. First, it was verified that the premises of dichotomy, multi-co-linearity, the Chi-square test, and mutual exclusivity held. To find potential variables for multivariable analysis, bi-variable analysis was used. In bi-variable logistic regression, a variable with a significant association was transferred to multivariable logistic regression. To assess the relationships between the outcome and explanatory factors, an adjusted odd ratio (AOR) with a 95% Confidence Interval (CI) and *p*-value was calculated. When it came to determining if an explanatory factor was substantially correlated with the outcome variable, a *p*-value of 0.05 was used. The multi-collinearity was examined using variance inflation factors (VIF), which should be less than 10. Hosmer and Lemeshow tests were performed to evaluate the goodness of fit, which was sig = 0.989. A histogram and Q–Q plot test were used to determine the normality of the data.

In the qualitative study, in-depth interviews and focused group discussions were used for data collection, while thematizing was used for analysis. From the collected data codes, categories and themes were extracted using atlas.ti.7.1. We (the primary investigators) transcribed verbatim from the recorded audio. Then we translated the transcribed data into English. Finally we presented the result by quotations derived from the data.

## 3. Results

### 3.1. Socio-demographic characteristics of the respondents

A sum of 422 women took part in the study, with a response rate of 100%. In total, 292 (69.2%) participants were between 20 and 30 years. A sum of 228 (54.0%) were Protestants, and 412 (97.6%) were married. For the level of education, only 78 (18.5%) had a college degree. The typical monthly household income in Ethiopian birr was between 2,000 and 4,000. Nearly half (259 (61.5%)) of the participants used taxis as their primary form of transportation to the medical facilities (Table 1).

**Table 1:** Socio-demographic characteristics of the respondents interviewed at Southwest Ethiopia Health Centers in 2022, (*n* = 422).

Variable	Characteristic	Respondents	
		Frequency (no.)	Percentage (%)
Age of the participant	</=19	8	1.9%
	20–30	292	69.2%
	31–40	103	24.4%
	41–50	19	4.5%
	Total	422	100%
Marital status of the participant	Married	412	97.6
	Divorced	–	0
	Widowed	10	2.4
	Total	422	100%
Educational status of the participant	No formal education	30	7.1
	Primary education	168	39.8
	High school and preparatory education	146	34.6
	Higher education	78	18.5
	Total	422	100
Educational status of the father	No formal education	8	1.9
	Primary education	108	25.6
	High school and preparatory education	76	18
	Higher education	230	54.5
Religion of the participant	Orthodox	116	27.5
	Protestant	228	54
	Others	22	5.2
	Muslim	56	13.3
Occupation of the participant	Employed	118	28
	Housewife (unemployed)	202	47.9
	Merchant	92	21.8
	Others	10	2.4
	Total	422	100
Family's monthly income	<2,000	150	35.5
	2,000–4,000	203	48.3
	>4,000	102	24.2
	Total	422	100
Means of transport to health facility	Public transport	259	61.4
	By foot	163	38.6
	Total	422	100

### 3.2. Reproductive characteristics of the participants

About 357 (84.6%) participants had no history of abortion. Approximately 262 (62.1%) participants were Para 2–4, and 312 (73.9%) currently had planned pregnancies. Furthermore, 317 (75%) participants undertook ANC follow-up once (Table 2).

**Table 2:** Reproductive characteristics of the participants at Southwest Ethiopia Health Centers in 2022, (*n* = 422).

Variable	Characteristic	Respondents	
		Frequency (no.)	Percent (%)
History of abortion	Yes	65	15.4
	No	357	84.6
	Total	422	100
Parity	Para 1	112	26.5
	Para 2–4	262	62.1
	≥5	48	11.4
	Total	422	100
Pregnancy status	Intended and supported	312	73.9
	Unintended but supported	100	23.7
	Unintended and unsupported	10	2.4
	Total	422	100
History of ANC	Yes	317	75
	No	105	25
	Total	422	100
Number of ANC visits (*n* = 317)	<4	149	47
	≥4	149	47
Gestational age of index pregnancy (*n* = 298)	<37 weeks	86	29
	≥37 weeks	212	71
Number of current pregnancy	Single	405	96
	Twins	17	4
Neonatal sex	Male	223	47.2
	Female	199	52.8
Neonatal feeding	Exclusive breastfeeding	327	77.5
	Formula feeding	95	22.5
APH during pregnancy	Yes	44	10.5
	No	378	89.5
HDP	Yes	25	5.9
	No	397	94.1
Premature rupture of membrane and choriamnioties	Present	11	2.5
	Absent	411	97.5

### 3.3. Obstetrics characteristics of the respondents

**Figure 1:**
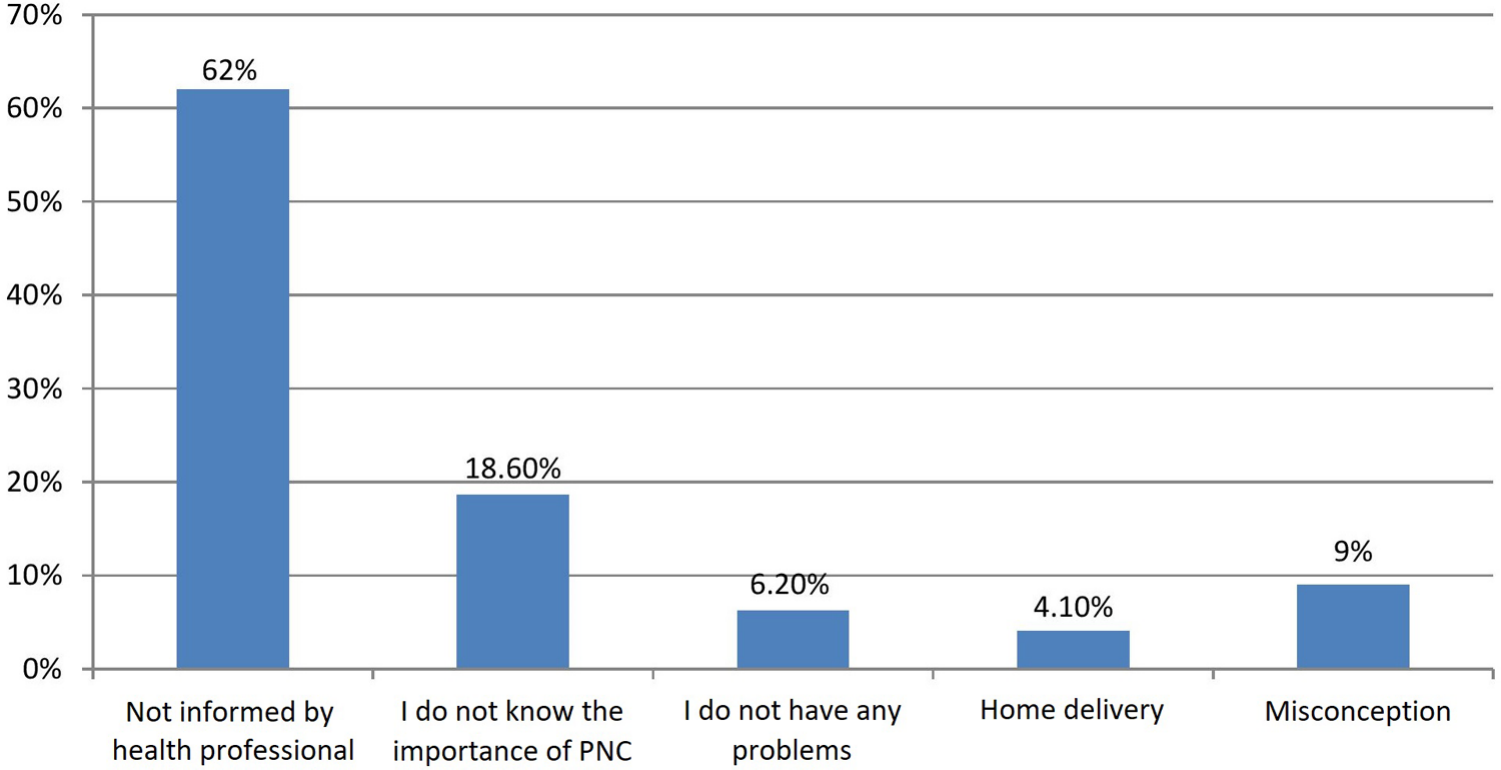
Reasons why the participants did not visit a health facility for postnatal care in Southwest Ethiopia Health Centers, 2022 (*n* = 422).

There were 287 (68%) institutional deliveries among the study's participants. The mode is 6 h, however the mean hospital stay before release after delivery was 26.7 h (+SD = 41 h). Approximately 304 (72%) of women stayed at a health institution for 6–11 h before discharge. About 252 (59.7%) of them were given appointments by health professionals for postnatal care before discharge. About 62 of them were not informed by health professionals about the accessibility of postnatal care. Out of those who knew about the availability of postnatal care, nearly 36% were not given appointments before discharge. Out of mothers who delivered in a health institution, 271 (67.2%) were counseled about danger signs that can happen during the postpartum period before discharge (Figure 1, Table 3).

**Table 3:** Obstetrics characteristics of the respondents interviewed at Southwest Ethiopia Health Centers in 2022, (*n* = 422).

Variable	Characteristic	Respondents	
		Frequency (no.)	Percent (%)
Delivery setting	Home	135	32
	Health center	185	43
	Private clinic	32	7.5
	Governmental hospital	70	17.5
Delivery attended by	Midwife (nurse)	202	48
	Physician	85	20
	Traditional birth attendant/family	135	32
Mode of delivery	Vaginal delivery	359	86.7
	Instrumental delivery	9	2.1
	Cesarean section	54	3.8
Minor inscision during vaginal delivery (*N* = 368)	Yes	97	26.4
	No	271	73.6
	Total	368	100
Cesarean delivery (*N* = 54)	Emergency	14	25.9
	Elective	40	74.1
	Total	54	100
Length of hospital stay following delivery	<6 h	304	72
	6–11 h	45	10.7
	12–23 h	54	12.8
	≥24 h	2	5
	Total	405	96
An appointment given for postnatal care	Yes	252	59.7
	No	152	36
	Total	404	95.7
Appointment within 72 h of discharge *N* = 252)	Yes	11	4.2
	No	241	95.6
Appointment within 1 week	Yes	26	10
	No	233	89.9
Appointment after 1 week	Yes	58	22.4
	No	201	77.6
Appointment after two weeks	Yes	27	10.4
	No	232	89.5
Appointment at 6 weeks	Yes	97	37.4
	No	162	62.5
Others	Yes	40	15.4
	No	219	84.5
Counsel for any danger signs before discharge	Yes	271	67.2
	No	132	32.7
	Total	403	95.5

### 3.4. Prevalence and characteristics of postnatal care utilization

The health center was the location most often used for postnatal care (177 (75.6%)), followed by government hospitals (38(16.2%)). The proportion of postnatal care visits within 72 h of discharge, one week, two weeks, and six weeks after delivery was 70 (29.9%), 92 (39.3%), 30(12.8%), and 25 (10.6%), respectively. Concerning the frequency of postnatal care visits, 79 (33.7%) participants had visited once, 60 (25.6%) twice, 66 (28.2%) thrice, and the remaining 26 (211.1%) had visited four times (Table 4).

**Table 4:** Characteristics of postnatal care utilization of interviewed respondents at Southwest Ethiopia Health Centers in 2022, (*n* = 422).

Variable	Characteristic	Respondents	
		Frequency (no.)	Percent (%)
Received postnatal care for current delivery	Yes	234	55.5
	No	188	44.5
Received postnatal care for the previous delivery	Yes	188	54.3
	No	158	45.7
Place where postpartum care was received	Health center	177	75.6
	Governmental hospital	38	16.2
	Private hospital	14	6
	Private clinic	5	2.1
Received postnatal care services within 72 h of discharge	Yes	70	29.9
	No	164	70.1
Received postnatal care within 1 week	Yes	92	39.3
	No	142	60.7
Received postnatal care within 2 weeks	Yes	30	12.8
	No	204	87.2
Received postnatal care at 6 weeks	Yes	25	10.6
	No	209	89.3
No. of postnatal care visits	Once	79	33.7
	Twice	60	25.6
	Thrice	66	28.2
	Fourth	26	11.1
Being with baby during postnatal care	Yes	220	94.
	No	14	6
Contraceptive use	Yes	273	64.7
	No	149	35.3
Contraceptive initiation period (*N* = 273)	After 6 weeks (42 days)	162	59.3
	After 2 months	50	18.3
	After 4 months	34	12.4
	After 6 months	27	9.8

### 3.5. Factors related to the use of postnatal care services

#### 3.5.1. Quantitative analysis

After conducting a bi-variable logistic regression analysis, *p*-values 0.25 were used to determine which variables should be included in a multivariable logistic regression. Out of 25 independent variables grouped under socio-demographic, reproductive and obstetrics, labor and postpartum characteristics, eight variables—educational status, pregnancy type, prior ANC use, delivery place, PNC appointment provided or not, healthcare practitioner counseling on danger signs given or not, prior history of PNC, and length of hospital stay before discharge—had shown association.

Three variables—counseling on PNC and appointment provision, health care practitioner counseling on danger signs, and prior history of PNC—showed a statistically significant association with PNC service use in multivariable logistic regression. As a result, women who received postnatal care counseling and appointments were roughly 3.6 [(AOR = 3.6, 95% CI (1.47–7.23)] times more likely to use PNC services than their counterparts. Women who received counseling from the healthcare professional on danger signs were twice as likely to receive a postnatal care visit than their counterparts [(AOR = 2, 95% CI (1.05–3.64)]. Similar to this, multiparous women who had previously used PNC services were three times more likely to do so for the current delivery than those who had not [AOR = 3, 95% CI (1.36–5.8)] (Table 5).

**Table 5:** Logistic regression analysis of factors associated with postnatal care utilization in the interviewed women.

Variable	Utilization of postnatal care		COR (CI)	AOR (CI)
Educational status	Yes	No		
No formal education	38	27	1.56 (0.57–4.4)	0.77 (0.39–1.52)
Primary education	99	59	1.88 (0.8–4.45)	0.92 (0.53–1.62)
High school and preparatory education	89	31	1.56 (0.85–2.9)	0.67 (0.92–5.36)
Higher education	51	28	1	1
Nature of pregnancy				
Intended and supported	213	98	2.61 (0.78–8.75)	1.0 (0.17–5.91)
Unintended but supported	59	41	1.73 (0.49–6.04)	0.6 (0.01–3.61)
Unintended and unsupported	5	6	1	1
Previous history of antenatal care				
Yes	273	139	5.93 (1.00–30.5)	3 (1.36–5.8)^a^
No	4	6	1	1
Delivery setting				
Home	2	14	0.07 (0.011–0.48)	0.31 (0.01–7.63)
Health center	168	139	1.62 (0.47–5.58)	1.01 (0.16–7.53)
Governmental hospital	66	57	0.58 (0.17–2.02)	0.59 (0.09–4.13)
Private hospital	33	18	0.92 (0.24–3.47)	0.74 (010–5.43)
Private clinic	8	4	1	1
Appointment provided for PNC and counseling				
Yes	266	55	3.95 (1.98–7.88)	3.6 (1.47–7.23)^a^
No	11	90	1	1
Counseling on danger signs				
Yes	202	56	4.28 (2.79–6.56)	2 (1.05–3.64)^a^
No	75	89	1	1
Length of stay at the health facility before discharge				
6–11 h	134	58	1.13 (0.69–1.83)	0.81 (0.36–1.83)
12–23 h	48	25	0.94 (0.51–1.72)	0.34 (0.13–0.88)
≥24 h	86	42	1	1

^a^ Significantly associated variables.

#### 3.5.2. Qualitative analysis

Qualitative data was obtained from the participants through in-depth interviews and focused group discussions to identify the barriers to PNC services utilization. The investigation revealed knowledge about postnatal care service utilization and access to health facilities were identified as barriers to postnatal care service utilization. The theme of knowledge included the sub-themes of ignorance, conventional wisdom, and religious activities. There were three sub-themes under access: waiting time, environmental exposure, and distance (Table 6).

**Table 6:** Barriers of postnatal care service utilization among women in Southwest Ethiopia Health facilities, 2022, (*n* = 422).

Major themes	Categories	Respondents
Lack of knowledge of postnatal care service utilization	Ignorance to health extension workers counseling about post natal care service utilization	Delivered mother
Traditional belief and religious activities	Conventional wisdom	Delivered mother
	Religious activity	Delivered mother
Lack of access to post natal care service utilization	Distances from delivered mother's home to health facilities	Delivered mother
	Waiting time at the health center	Delivered mother
	Environmental exposure such as sunlight, wind, and rain	Delivered mother

**Lack of knowledge about postnatal care service utilization**

1. Ignorance

Mothers, husbands, and other members of families discussed that they ignore education and advice on the importance of postnatal care service utilization from the health extension worker at the community level. They stated that even though health extension workers call them for meetings, they are busy with their farm work:

“*I do not believe that education on postnatal care service utilization by health extension workers is important to me because before I gave birth I had no time to spend with them. Sometimes I had time, but I forgot the call from the health extension worker and wasn’t available at the kebele’s clinic. After I gave birth, [the health worker] visited us and told me that I had to visit the health center, but I was fine and didn’t accept her counseling*” (30-year-old delivered mother).

**Traditional belief and religious activities**

1. Conventional wisdom

Conventional wisdom is another barrier mentioned by the study participants. They stated that going out of the house before forty days of delivery exposed the newborn and mother to evil spirits:

“*Even though those health professionals tell us to visit a health center after we give birth, we can’t go out of our home because the evil spirit will attack us and our newborn may be exposed to individuals with an evil eye. We can walk around our compound with a member of our family holding a knife because the evil spirit will not approach us while we hold a knife*” (34-year-old delivered mother).

2. Religious activities

Concerning a religious conviction, the study participants learned that among the community members who practiced Ethiopian orthodox Christianity, it was forbidden for the mothers and neonates to leave the house prior to the date of baptism, out of fear of an evil spirit. As a result, they claimed that they had not visited the health facilities for PNC services before the date of baptism:

“*On the 45th day after I gave birth, the health extension worker came to see us the day before yesterday. This is due to the cultural prohibition on leaving the house prior to the date of baptism*” (28-year-old female IDI participant).

Another idea mentioned by research participants was “hamechisa” (a festival in which newborns are taken to be blessed by a traditional healer, i.e., a witch), which takes place within the first two months of life. It was alleged that community members who followed this tradition did not go to the health facilities to seek any medical care before infants were blessed by the witch. They thought that unless the new-borns were seized and blessed, the witch or traditional healers would curse the family—especially the recently delivered mother and infant—and seriously implore a supernatural force to hurt or kill them:

“*It is our ancestors’ culture we inherited; I have to allow the witch or healer in our kebele because if I don’t do that, it is dangerous for me and my infant*” (25-year-old delivered woman).

**Access to health care facilities for postnatal care**

1. Distances

Some of the health professionals reported that most of the mothers are far from the health facilities and that the road to their homes is so dangerous that available means of transportation, even ambulances, face challenges serving them. Furthermore, they explained that even during childbirth, the families and neighbors of the women carry the woman to the health facilities, and this takes a long time. For example, one of the mothers said:

“*My home is so far away from a health facility that I didn’t visit a health facility. Even though I gave birth at the health facility, there was no means of transportation other than parts of my family and neighbors who carried me to health facilities. Therefore, I didn’t visit a health facility because of the distance after I gave birth*” (35-year-old delivered mother).

2. Environmental exposures

Most of the participants mentioned that environmental factors, including cold weather conditions, sunlight, and rain, are commonly known factors that prohibit mothers from visiting health facilities during PNC. One of the participants said:

“*After we give birth, it is very difficult to resist sunlight, cold weather, and rain because our body is too weak and fragile. Therefore, immediately after we gave birth, we had to stay in a safe environment, and that is our home*” (30-year-old delivered mother).

Other participants also reported that they could not afford to buy protective materials from sunlight and rain, such as an umbrella. For instance, other women said:

“*In fact, an umbrella is preferred during rain and sunlight, but I can’t afford to buy it. I had one, but it was broken, and I couldn’t buy a new one. Therefore, it is better to stay home than walk in the sun, light, and rain without an umbrella*” (29-year-old delivered mother).

3. Waiting time at the health center

Other participants complained that they had to wait a long time to receive care after they reached the health facilities during postnatal care. The following participant said:

“*The last year before I got pregnant, we visited health facilities with my brother’s wife after she gave birth. I remember we stayed for many hours to get our turn to receive the care. Therefore, I didn’t visit the health facility after I gave birth because I remembered how long we waited to get our turn for receiving care last year*” (31-year-old delivered mother).

## 4. Discussion

This study evaluated factors and barriers related to postnatal care service use among women who delivered a baby and received vaccinations. Despite nearly 287 (68%) of the participants in this study having given birth in a medical facility, it was found that just over half of them, or 234 (55.5%), had a checkup following delivery. This number, however, is greater than the 2019 EDHS postnatal care use rate of 48% (4). The time gap and various strategies for obtaining and utilizing maternal healthcare services may be responsible for this improvement.

The prevalence of postnatal care utilization found in this study was also higher than in studies similar to this one that were carried out in Enderta District, Tigray (49.7%), Jabitena District, Amhara Region (20.2%), Southern Ethiopia (37.2%), Hawassa Zuria District, Southern Ethiopia (37.2%), Adigirat Town, Northern Ethiopia (34.3%), Northern Ethiopia (37%), and East Gojam, Northwest Ethiopia (12, 14, 19, 21–23, 25, 29, 30). The demographic homogeneity variation in the research areas may be related to the inconsistencies. The results of this study were also slightly higher than those of research carried out in Burkina Faso (44%), Cambodia (46%), Haiti (55%), Kenya (46%), Malawi (41%), Mali (49%), Nigeria (46.5%), and Zambia (41%), respectively, across Africa and Asia (20). The results, however, fell short of those of studies carried out in the Ethiopian towns of Debre Tabor (57.5%), Addis Ababa (65.6%), and Sodo Zuria (77.7%). The study results from Ethiopia's Shebe Sombo Woreda, Jima Zone (58.5%), Brazil (77%), Bangladesh (73%), Uganda (57%), Nepal (72%), and Rwanda (71%) all showed comparable differences. This implies that the utilization of postnatal care in this study area is considerably lower compared with other lower and middle income countries. This might be due to poor maternal care quality of services in the country which include a lack of counseling services on postnatal care service utilization and danger signs after delivery. Therefore all concerned stakeholders should work in collaboration to increase postnatal care service utilization uptake (9, 10, 19, 27, 28).

In our study, the health provider's PNC counseling and appointments, the counseling of women on danger signs, and a history of prior PNC use were the most related factors for PNC use. In comparison to their counterparts, women who received counseling and appointments for postnatal care services were roughly 3.6 times more likely to use the PNC service [AOR = 3.6, 95% CI (1.47–7.23)]. An investigation carried out in Northern Ethiopia and Shebe Sombo Woreda, Jima Zone, Ethiopia, supports this conclusion (12, 29). This finding would suggest that women's knowledge of the advantages of postnatal care significantly affects their use of PNC services. This finding implies that postnatal care appointments should be incorporated to optimize postnatal care uptake.

Counseling women about danger signs during the postnatal period was the other factor linked to the use of postnatal care services. Women who received information about any postpartum warning symptoms were twice as likely to use postpartum care services than their counterparts [AOR = 2, 95% CI (1.05–3.64)]. This result is consistent with a study carried out in Southern Ethiopia's Amhara Region and Hawassa Zuria District, which found that mothers who were aware of at least one postpartum obstetric danger sign were more likely to use PNC services than those who were not (12, 30). This result is also supported by investigations carried out in Uganda and Nepal that produced comparable results (15, 16). This similarity can be explained by the fact that knowledge of obstetric danger signs is a significant motivator for encouraging women and their families to seek medical attention as soon as possible in order to prevent, identify, and manage their obstetric danger symptoms. The implication of this result shows that all mothers and their families ought to be mindful of danger signs amid the postnatal period. Health professionals should audit the emergency plans made amid the postnatal period to see whether they are still suitable. Health professionals should also remind mothers to bring their maternal wellbeing record with them even for emergency visits, as it is important in the optimization of postnatal care service utilization.

Utilization of postnatal care services in the past was the other key factor associated with postnatal care service use. Women who had previously given birth and had PNC were three times more likely to now seek PNC services than those who had never used PNC services in the past [AOR = 3, 95% CI (1.36–5.78)]. This strong positive association between PNC service utilization and prior history can be attributed to the fact that women who had PNC in health institutions had a greater opportunity to be exposed to health education related to PNC services at the time of their visit. Past experiences regarding PNC are essential to improve the quality of essential, routine postnatal care for women and newborns by targeting a positive postnatal experience.

Last but not least, unlike earlier studies in Ethiopia (12–30) which discovered that the participants' socioeconomic status, ANC, and educational status were the drivers of PNC utilization, these characteristics and others were not. This might be the case since most women have similar access to information on PNC service utilization through the media or at their ANC follow-up visits, and the study was carried out in the most urban areas of the nation.

In the qualitative analysis, similar to the study done in East Gojam Zone in Ethiopia and Debre Libanos District in Ethiopia (17, 18), the knowledge theme, which includes the sub-themes of ignorance, conventional wisdom, and religious activities, was identified as a barrier to the use of postnatal care. This might be the result of the cultural and religious similarity of the study population. It also emphasizes the significance of social and behavioral change and communication to alter community members' or religion leaders' perceptions and foster the development of health-seeking behavior such as employing PNC services. Furthermore, sub-themes of access, which include waiting time, exposure to the environment, and distance, were determined to be barriers to the use of postnatal care. This finding is also supported by research done in the East Gojam Zone in Ethiopia and Debre Libanos District in Ethiopia (17, 18). This might be the result of the similarity of the weather conditions, health care facility service provision, and distribution. To reduce these barriers and optimize the uptake of postnatal care an innovative approach to increase the health literacy on postnatal care is required. Furthermore, increasing the number of health facilities is mandatory to alleviate these barriers.

## 5. Conclusion

According to this study, overall use of postnatal care was still low and there is much room for improvement. There are various factors and barriers associated with postnatal service utilization. Therefore, all stakeholders should consider these factors to optimize the use of postnatal care.

## Data Availability

The original contributions presented in the study are included in the article/Supplementary Material, further inquiries can be directed to the corresponding author.
